# Smart Device-Supported BDS/GNSS Real-Time Kinematic Positioning for Sub-Meter-Level Accuracy in Urban Location-Based Services

**DOI:** 10.3390/s16122201

**Published:** 2016-12-21

**Authors:** Liang Wang, Zishen Li, Jiaojiao Zhao, Kai Zhou, Zhiyu Wang, Hong Yuan

**Affiliations:** 1 Academy of Opto-Electronics, Chinese Academy of Sciences, Beijing 100094, China; zhaojj@aoe.ac.cn (J.Z.); zhoukai@aoe.ac.cn (K.Z.); wangzhiyu15@mails.ucas.ac.cn (Z.W.); yuanh@aoe.ac.cn (H.Y.); 2 Key Laboratory for Urban Geomatics of National Administration of Surveying, Mapping and Geoinformation, Beijing 100044, China; 3 University of Chinese Academy of Sciences, Beijing 100049, China

**Keywords:** global navigation satellite system (GNSS), BeiDou navigation satellite system (BDS), real-time kinematic (RTK), smart devices, location-based services (LBS)

## Abstract

Using mobile smart devices to provide urban location-based services (LBS) with sub-meter-level accuracy (around 0.5 m) is a major application field for future global navigation satellite system (GNSS) development. Real-time kinematic (RTK) positioning, which is a widely used GNSS-based positioning approach, can improve the accuracy from about 10–20 m (achieved by the standard positioning services) to about 3–5 cm based on the geodetic receivers. In using the smart devices to achieve positioning with sub-meter-level accuracy, a feasible solution of combining the low-cost GNSS module and the smart device is proposed in this work and a user-side GNSS RTK positioning software was developed from scratch based on the Android platform. Its real-time positioning performance was validated by BeiDou Navigation Satellite System/Global Positioning System (BDS/GPS) combined RTK positioning under the conditions of a static and kinematic (the velocity of the rover was 50–80 km/h) mode in a real urban environment with a SAMSUNG Galaxy A7 smartphone. The results show that the fixed-rates of ambiguity resolution (the proportion of epochs of ambiguities fixed) for BDS/GPS combined RTK in the static and kinematic tests were about 97% and 90%, respectively, and the average positioning accuracies (RMS) were better than 0.15 m (horizontal) and 0.25 m (vertical) for the static test, and 0.30 m (horizontal) and 0.45 m (vertical) for the kinematic test.

## 1. Introduction

With the development of global navigation satellite systems (GNSSs), the location-based services (LBS) industry has become one of the fastest-growing emerging information industries [1]. Smart devices, which are usually based on Android or iOS platforms, are being widely used due to the booming development of mobile Internet technology and they are playing important roles in LBS. Achieving positioning and navigation with sub-meter-level accuracy on smart devices is an important application field for the future development of GNSSs. Moreover, the multi-GNSS combined positioning, which can significantly improve the positioning availability, continuity and accuracy, has become an inevitable trend in future location-based applications [2,3]. Currently, with the operation of the Chinese BeiDou Navigation Satellite System (BDS) [4] in the Asia-Pacific region, excellent opportunities for BDS/GNSS combined positioning are available for users [2,5,6]. Precise positioning and navigation based on low-cost receivers also becomes possible with the support of multi-constellations [7,8,9,10]. However, the positioning accuracy provided by the commonly used smart devices, such as smartphones with an embedded GNSS chip, is only about 15–20 m through the single-point positioning (SPP) approach [11], and it is very difficult to meet the demands of high-precision LBS, which usually requires sub-meter-level positioning [12,13].

Compared with SPP, the differential positioning approach, which is implemented based on the differential corrections from nearby reference stations, is still one of the commonly used techniques for achieving high-precision and instantaneous positioning in practice. The differential positioning technique can be divided into two categories, i.e., DGNSS (differential GNSS) and RTK (real-time kinematic). The former is usually implemented using the code observables, whereas the latter uses the carrier phase. Previous studies [5,14,15] show that the positioning accuracies of DGNSS and RTK with a geodetic receiver are about 1.0–2.0 m and 0.02–0.05 m, respectively. Due to the high price and large size, the geodetic receivers cannot be directly used in LBS services. Different from the geodetic receivers which can generate the code and carrier phase observables with high precision, the observables from those mass-market navigational receivers generally have low precision [16,17]. As a result, the accuracy of DGNSS with a low-price navigation terminal can only reach about 3.0–5.0 m and RTK positioning is very difficult to achieve [17]. In recent years, several novel PPP (precise point positioning)-RTK positioning methods have been proposed and studied [18,19,20,21,22,23,24]. For implementing PPP-RTK positioning, the un-differenced ambiguities of the carrier phase need to be fixed at a single receiver. This can be achieved by correcting the fractional cycle biases which are estimated based on the observation data from a station network and broadcasted to users as products. However, since the data quality of a low-cost receiver is poorer than that of the commonly used geodetic receiver, the performances of un-differenced integer ambiguity resolution and PPP-RTK positioning in practice still need to be further studied.

In order to further improve the positioning performance of smart devices, an approach for combining the smart device and the low-cost GNSS module which can provide the code and carrier phase observables is proposed in this study to achieve a high-accuracy positioning with the RTK technique, as well as the software of BDS/GNSS RTK positioning installed on the Android platform. The proposed approach is a feasible solution to achieve high-accuracy positioning with such a smart device which cannot provide the observables with high precision. The performances of the proposed approach and developed software were validated by real-time static and kinematic tests in a real urban environment under the BDS and GPS constellations.

This paper is organized as follows: first, the system design scheme and implementation solutions for realizing RTK positioning based on smart devices are described in Section 2; secondly, the experimental results of the performance validation in a real urban environment are given in Section 3; and, finally, the conclusions are drawn and future works are discussed in Section 4.

## 2. System Design Scheme and Implementation

### 2.1. System Design Scheme

RTK positioning requires not only the raw observations and satellite ephemeris of the rover, but also the raw observations or the differential corrections from nearby reference stations. The communication link or network for data transmission between the reference station and the rover is also needed in RTK positioning.

It is very easy to collect the required raw observations and ephemeris by a general GNSS receiver, but it is not feasible for most smart devices with an embedded GNSS chip (such as smartphones, etc.) [11,25]. Therefore, obtaining the required observations is a prerequisite for achieving RTK positioning on smart devices. To solve this problem, an effective way is using an external GNSS module (such as u-blox, etc.) to provide the required rover observations and ephemeris and collecting these data in real-time using those smart devices with the built-in Bluetooth modules via the Bluetooth protocol. With respect to the data transmission from the reference station to the rover, specialized equipment, such as a radio frequency station, is generally used in traditional RTK applications. However, it is not well-suited for mass-market LBS applications. Since smart devices are usually equipped with Internet-accessing capabilities, the communication problem can be easily solved by accessing the 3G/4G cellular or Wi-Fi (if available) network.

**Figure 1:**
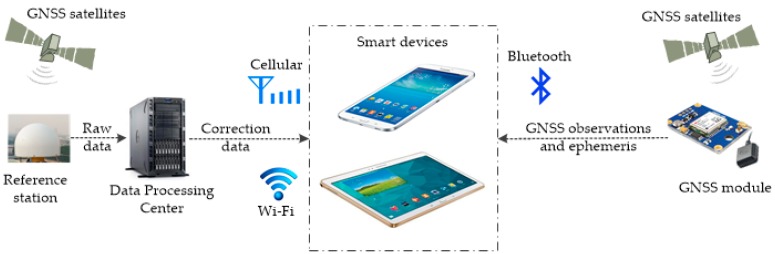
Framework of an RTK service system based on smart devices.

The main integration idea for smart device–supported RTK positioning is making the most of the wireless communication ability, the computing capability, and the extensive-usage advantage of those mass-market smart devices. In this contribution, a real-time RTK service system was designed for precise LBS, and the precise LBS services can be further extended (e.g., the lane-level navigation) once the user has the ability to obtain high-accuracy positioning results. This system mainly consists of a reference station, a data processing center (DPC), and a user part, as shown in Figure 1.

The reference station equipped with a high-performance geodetic receiver is for collecting the raw GNSS observations, including the measurements of the code and carrier phase and the satellites’ ephemeris, etc., and sending them to the DPC in real time through a data communication link, such as the Internet used in our system. The sampling rate of the raw observations at the reference station is 1 Hz.

The functions of the DPC consist of receiving the data streams from the reference station in real time, calculating the differential correction information and broadcasting them to users through a wireless network. In our system, the differential corrections are applied instead of sending the raw observations for reducing the amount of data to be transmitted and the cost of communication. They are calculated by the DPC with a specific algorithm and broadcasted to users through the user datagram protocol (UDP).

The user part includes an external GNSS module and a smart device, e.g., a smartphone. The main work of the user part is providing the real-time high-accuracy results by RTK positioning. The smart device collects the observation and ephemeris data (in binary) provided by the external GNSS device through its built-in Bluetooth module. Meanwhile, it establishes a communication connection with the DPC and receives the differential corrections via a 3G/4G cellular or Wi-Fi (if available) network using the UDP protocol. Thus, the RTK positioning can be achieved with the raw observations, ephemeris, and differential corrections. If the network communication with the DPC is lost, the single-point positioning solutions will be given with only the raw observations being used, and if the Bluetooth connection with the GNSS module is temporarily lost, the self-positioning results of the smart device (from the embedded GNSS chip) will be returned to the user.

With respect to the smart devices, there are no other special requirements for them in this system. The basic requirements are that they: (1) run Android OS 4.1 or above; (2) have a built-in Bluetooth module (version 4.0 will be better) for collecting the raw data stream from the external GNSS module; and (3) have Internet accessibility (cellular or Wi-Fi networks are both okay) for receiving the differential correction data.

### 2.2. Mathematical Model

The double-differenced (DD) observation model is widely used in RTK positioning. It can eliminate the satellite and receiver clock errors and significantly mitigate the atmospheric (ionosphere and troposphere) delay errors under the condition of a short baseline [14,26], especially when the ionosphere is quiet [27]; therefore, it is beneficial for ambiguity resolution. The basic non-linear code and carrier phase observation equation of the un-differenced (UD) model on one frequency is given in Equation (1):

(1) $$\left\{ \begin{array}{ll} P_{r}^{j} & =\rho_{r}^{j}+c(dt_{r}-dt^{j})+I_{r}^{j}+T_{r}^{j}+\varepsilon_{r}^{j}\\ \lambda \phi_{r}^{j} & =\rho_{r}^{j}+c(dt_{r}-dt^{j})-I_{r}^{j}+T_{r}^{j}+\lambda N_{r}^{j}+\varsigma_{r}^{j} \end{array}\right.$$

where *P* is the code observation (meters); *φ* is the carrier phase observation (cycle); *j* and *r* represent the satellite and receiver, respectively; *ρ* is the geometric distance from the satellite to the receiver; *c* is the velocity of light; *dt^j^* and *dt_r_* denote the clock error of the satellite *j* and the receiver *r*, respectively; *I* and *T* are the ionospheric and tropospheric delays, respectively; *λ* is the wavelength; *N* is the phase ambiguity (cycle); *ε* and *ζ* represent the code and carrier phase observation noise, including some unmodeled errors, e.g., multipath, respectively.

At the reference station *r*, the geometric distance $\rho_{r}^{j}$ from the satellite *j* to the receiver antenna can be derived as Equation (2):

(2) $$\rho_{r}^{j}=\sqrt{{\left(X^{j}-X_{r}\right)}^{2}+{\left(Y^{j}-Y_{r}\right)}^{2}+{\left(Z^{j}-Z_{r}\right)}^{2}}$$

where, [*X^j^, Y^j^, Z^j^*] denotes the position of satellite *j* which can be calculated from the broadcast ephemeris; [*X_r_, Y_r_, Z_r_*] denotes the position of reference station *r* which is usually known. Thus, the differential correction of code $\delta P_{r}^{j}$ and carrier phase $\delta \phi_{r}^{j}$ can be calculated by Equation (3) and they will be broadcasted to users by the communication link established in the real-time RTK service system:

(3) $$\left\{ \begin{array}{l} \delta P_{r}^{j}\;=P_{r}^{j}-\rho_{r}^{j}=c(dt_{r}-dt^{j})+I_{r}^{j}+T_{r}^{j}+\varepsilon_{r}^{j}\\ \delta \lambda \phi_{r}^{j}=\lambda \phi_{r}^{j}-\rho_{r}^{j}=c(dt_{r}-dt^{j})-I_{r}^{j}+T_{r}^{j}+\lambda N_{r}^{j}+\varsigma_{r}^{j} \end{array}\right.$$

Hence, for the user *u*, the corrected code $\Delta P_{ur}^{j}$ and carrier phase $\Delta \phi_{ur}^{j}$ can be calculated in Equation (4) which is the single-differenced (SD) model between two receivers:

(4) $$\left\{ \begin{array}{l} \Delta P_{ur}^{j}\;=P_{u}^{j}-\delta P_{r}^{j}\\ \;=\rho_{u}^{j}+c(dt_{u}-dt_{r})+(I_{u}^{j}-I_{r}^{j})+(T_{u}^{j}-T_{r}^{j})+(\varepsilon_{u}^{j}-\varepsilon_{r}^{j})\\ \;=\rho_{u}^{j}+cdt_{ur}+I_{ur}^{j}+T_{ur}^{j}+\varepsilon_{ur}^{j}\\ \Delta \lambda \phi_{ur}^{j}=\lambda \phi_{u}^{j}-\delta \lambda \phi_{r}^{j}\\ \;=\rho_{u}^{j}+c(dt_{u}-dt_{r})-(I_{u}^{j}-I_{r}^{j})+(T_{u}^{j}-T_{r}^{j})+\lambda (N_{u}^{j}-N_{r}^{j})+(\varsigma_{u}^{j}-\varsigma_{r}^{j})\\ \;=\rho_{u}^{j}+cdt_{ur}-I_{ur}^{j}+T_{ur}^{j}+\lambda N_{ur}^{j}+\varsigma_{ur}^{j} \end{array}\right.$$

Then, choosing the highest-elevation satellite (referred to as *k*) as the reference satellite to form the double-differenced (DD) model, as shown in Equation (5):

(5) $$\left\{ \begin{array}{l} \nabla \Delta P_{ur}^{jk}\;=\Delta P_{ur}^{j}-\Delta P_{ur}^{k}\\ \;=(\rho_{u}^{j}-\rho_{u}^{k})+(I_{ur}^{j}-I_{ur}^{k})+(T_{ur}^{j}-T_{ur}^{k})+(\varepsilon_{ur}^{j}-\varepsilon_{ur}^{k})\\ \;=\rho_{u}^{jk}+I_{ur}^{jk}+I_{ur}^{jk}+\varepsilon_{ur}^{jk}\\ \nabla \Delta \lambda \phi_{ur}^{jk}=\Delta \lambda \phi_{ur}^{j}-\Delta \lambda \phi_{ur}^{k}\\ \;=(\rho_{u}^{j}-\rho_{u}^{k})-(I_{ur}^{j}-I_{ur}^{k})+(T_{ur}^{j}-T_{ur}^{k})+\lambda (N_{ur}^{j}-N_{ur}^{k})+(\varsigma_{ur}^{j}-\varsigma_{ur}^{k})\\ \;=\rho_{u}^{jk}-I_{ur}^{jk}+T_{ur}^{jk}+\lambda N_{ur}^{jk}+\varsigma_{ur}^{jk} \end{array}\right.$$

For the short-baseline conditions, the following approximations can be accepted:

(6) $$\left\{ \begin{array}{l} I_{u}^{j}\approx I_{r}^{j},T_{u}^{j}\approx T_{r}^{j}\\ I_{ur}^{jk}\approx 0,T_{ur}^{jk}\approx 0 \end{array}\right.$$

Thus, from Equations (5) and (6) it can be seen that the satellite and receiver clock errors are eliminated and the atmospheric (ionosphere and troposphere) delay errors are mitigated significantly under the condition of a short baseline.

In addition, the stochastic model is essential for obtaining high-precision positioning results and the elevation-dependent weighting model [28] is adopted in the proposed approach, shown in Equation (7):

(7) $${\left(\sigma_{r}^{j}\right)}^{2}=a^{2}+b^{2}\cdot f^{2}(el){|}_{r}^{j}$$

where ${\left(\sigma_{r}^{j}\right)}^{2}$ is the variance of the un-differenced observation; *r* represents the receiver; *j* represents the visible satellite; *a* and *b* are the constant error factors which are usually chosen empirically or calculated with the prior information of the observed data; *el* denotes the elevation angle from the receiver to the observed satellite; and *f*(*el*) = 1/sin(*el*) [28] is an elevation-related function.

### 2.3. Ambiguity Resolution and Validation

Compared with the positioning results derived from the float or incorrectly fixed ambiguities, the positioning accuracy can be improved when the ambiguities can be correctly fixed. In the proposed approach, the Least-squares AMBiguity Decorrelation Adjustment (LAMBDA) method [29,30] was applied for ambiguity resolution to obtain the integer ambiguities (fixed solution) after obtaining the float estimates of the ambiguities. For validating the integer ambiguity estimates, there are two widely used methods, i.e., the traditional ratio test (RT) and the fixed failure-rate ratio test (FF-RT) [31,32,33,34,35,36]. The traditional RT approach adopts a fixed critical threshold which is usually chosen from experience, e.g., 1.5, 2, or 3 [26,37]. The threshold with an improper selection would largely affect the positioning results. A high threshold may lead to a large probability of “false alarms” (i.e., refusing the correctly fixed integer ambiguities), while a low threshold may result in a higher probability of “missed detection” (i.e., accepting the incorrectly fixed integer ambiguities). The FF-RT approach is a detection criteria based on a fixed failure rate (*P_f_*) of which the critical threshold is determined according to the user-defined acceptable failure rate, the number of ambiguities, and the conditional variance of the ambiguities. Section 2.4.2 shows a comparison of the effects on the performance of RTK positioning based on the methods of RT and FF-RT as an example. In this contribution, the FF-RT method was used for validating the fixed ambiguities and the acceptable failure rate was set to *P_f_* = 0.01.

### 2.4. Latency Time of the Differential Corrections

In the RTK system, the differential corrections calculated from the observations of the reference station are usually sent to users through a wireless communication link. As a result, it is impossible to avoid the latency time in receiving the corrections and even the temporary interruption due to the failure of the network connection. Since the latency time and temporary interruption are generally dependent on the performance of the wireless link [38], there are two problems that need to be considered: (1) the length of the latency time of the differential corrections; and (2) the effect of the latency time (includes the temporary interruption) on the ambiguity resolution and positioning accuracy. The above two problems are analyzed by the following tests, respectively.

#### 2.4.1. Analysis of the Latency Time of the Differential Corrections

This test included two steps. First, the time of the DPC center server and the user-side smart device were synchronized and maintained through the network time protocol (NTP). Secondly, the DPC server sent the correction packets to the user with a transmission timestamp and the user marked a reception timestamp for the differential correction packets received. After a continuous test for about 12 h with a Wi-Fi network connection, the length of the latency time can be obtained by calculating the time differences between the transmission time and the reception time.

**Figure 2:**
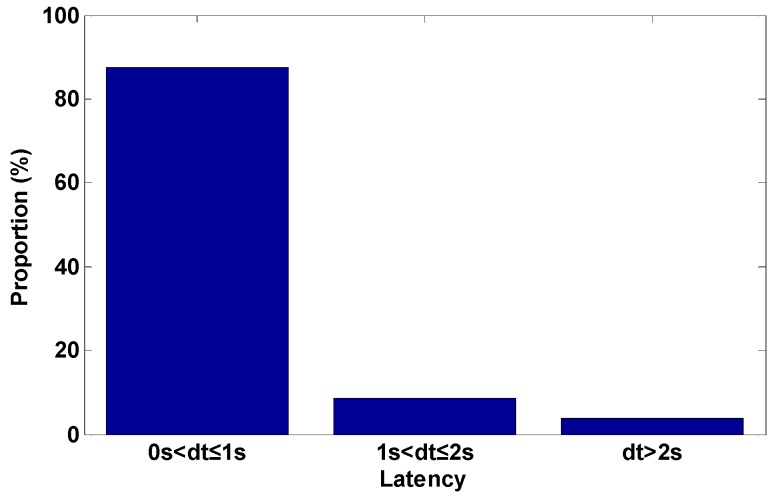
The statistical results of the length of the latency time of the corrections.

Figure 2 shows the statistical result of the length of the latency time of the corrections. We can find that the latency time of the differential corrections in the communication was almost within two seconds. It should be noted that the corresponding latency time of differential corrections may be slightly different, depending on the performance of the communication networks accessed in the practical application.

#### 2.4.2. Analysis of the Effect of Latency Time on RTK Positioning

To reduce the effect of the latency time of the differential corrections on a user’s positioning performance, the variation rate of the code and carrier phase correction is also included in the differential correction information [39]. However, with the increase of the latency time of the corrections, especially the temporary interruption of communication, the RTK positioning performance will gradually become worse. In order to analyze the effect of the latency time on the performance of ambiguity resolution and positioning accuracy with RTK, a test including three types of latency time of the differential corrections, i.e., 0 s, 5 s, and 10 s, was carried out by post-processing RTK positioning. Moreover, the difference between the traditional RT and FF-RT was also tested with the consideration of different latency times of the differential corrections. The data used in this test was collected in static mode under the condition of a short baseline by two GPS/BDS receivers with a testing period of about 15 h, and the distance between the reference station and the rover was about 5 km. The test results are shown in Table 1.

Here it is worth noting that the whole positioning solutions in this contribution were divided into two types: the fixed solutions and the unfixed solutions. A “fixed solution” means that the positioning result was obtained with the ambiguity parameters being fixed as integers, while an “unfixed solution” is just derived from a failed ambiguity resolution result or by single-point positioning when the differential corrections are not available. The “whole solutions” are the combination of the fixed solutions and the unfixed solutions during the whole testing period. The definition of “fixed-rate” in this contribution implies the proportion of those epochs where the ambiguities are fixed during the whole testing period, which was calculated by Equation (8):

(8) $$fixed-rate=\frac{\#\;of\;fixed\;epochs}{\#\;of\;total\;epochs\;}$$

Additionally, the root mean square (RMS) errors shown in Table 1 were computed for the three-dimensional (3D) position in relation to the precise benchmark coordinates.

It can be seen from Table 1 that the performances of ambiguity resolution and positioning accuracy are gradually decreased with the increase of the latency time for both ambiguity validation approaches (traditional RT and FF-RT). The deterioration of the positioning performance under the conditions of such latency times is tolerable for the demands of sub-meter-level accuracy. In addition, compared with the results based on the traditional RT with a fixed threshold, although the accuracy of the fixed solutions based on the FF-RT approach is a little poorer, its ambiguity fixed rate and positioning accuracy of the whole solutions are better. For the real-time navigation applications, therefore, the RTK positioning based on FF-RT can obtain a relatively better positioning performance both in accuracy and reliability, which can effectively meet the sub-meter accuracy demands for urban location-based services.

### 2.5. Software Implementation

**Figure 3:**
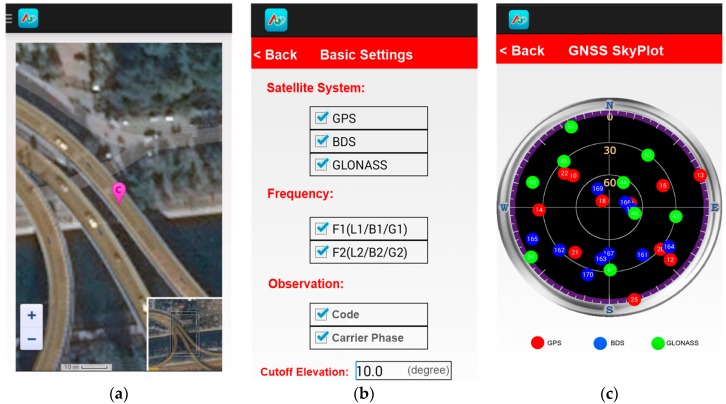
Operation interfaces of user-side positioning software: (**a**) the interface for displaying the user’s position on a map; (**b**) the interface for setting the positioning parameters; and (**c**) the interface for the current sky plot of visible satellites.

Based on the aforementioned approaches, a set of software for realizing BDS/GNSS real-time RTK services was developed from scratch, including the data processing software for DPC based on the Windows platform and the RTK positioning software running on smart devices based on the Android platform. Currently, this system can support real-time BDS, GPS, and GLONASS data processing and RTK positioning. Figure 3 shows some operation interfaces of the application software on the user side.

## 3. Experiments and Results

### 3.1. Outline of the Experiments

**Figure 4:**
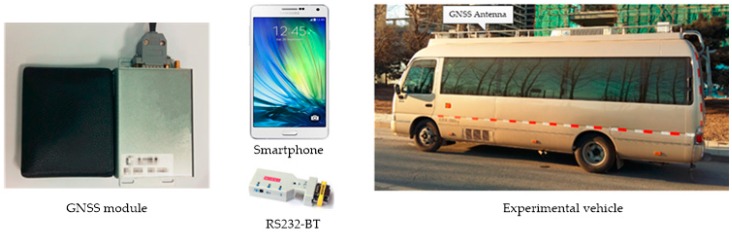
The experimental setup.

In order to validate the performance of smart device–supported RTK positioning, both the static and kinematic tests were carried out in a real urban environment in Beijing, and each test included three cases. The reference station (named AOE) was installed on the roof of the building of the Academy of Opto-Electronics in Beijing with good observation conditions. The GNSS receiver used on the user-side was an OEM615 navigation module manufactured by the NovAtel Company, Canada. Figure 4 shows the experimental setup, including the appearance of the GNSS module (wallet-size). A Samsung Galaxy A7 smartphone was used in the tests for the data collection of the raw observations, ephemeris and corrections and the calculation of RTK positioning. The communication network accessed was the 3G/4G cellular communication network operated by the China Mobile Communications Corporation. The RS232-BT module was connected with the GNSS module for sending the raw GNSS data to the smartphone through the Bluetooth protocol. The devices used in the tests cost less than $1000 USD in total.

In addition, in order to validate the positioning accuracy of the user’s real-time positioning results, the “true” position (with an accuracy of better than 5 cm) of the user for comparison was obtained by a high-performance GPS/INS (inertial navigation system) integrated receiver (installed in the experimental vehicle shown in Figure 4) which shared a common antenna with the user receiver via a power divider. The GNSS antenna was installed on the experimental vehicle, as shown in Figure 4. The positioning mode in all experiments was BDS/GPS combined single-epoch RTK positioning and the sampling rate of observation was 1 Hz. The cutoff elevation angle was set to 15 degrees and the constant error factors (*a*, *b*) for the stochastic model in Equation (7) were set as Equation (9), and the acceptable failure rate was set to *P_f_* = 0.01 for FF-RT validation.

(9) $$a=b=\left\{ \begin{array}{ll} 0.3\;m, & \mathrm{for}\;\mathrm{code}\\ 0.005\;m, & \mathrm{for}\text{ carrier-phase} \end{array}\right.$$

**Figure 5:**
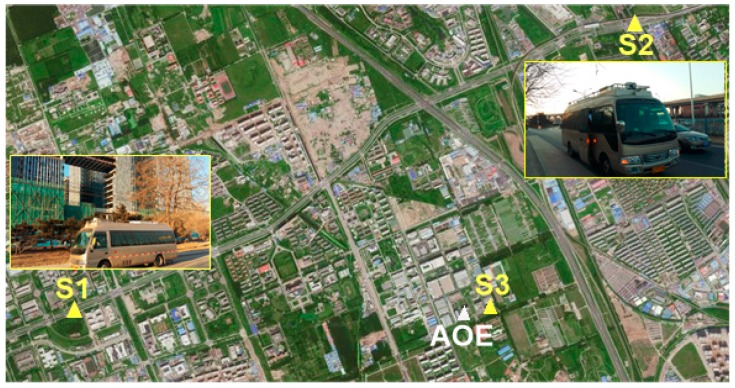
User sites in the static tests.

**Figure 6:**
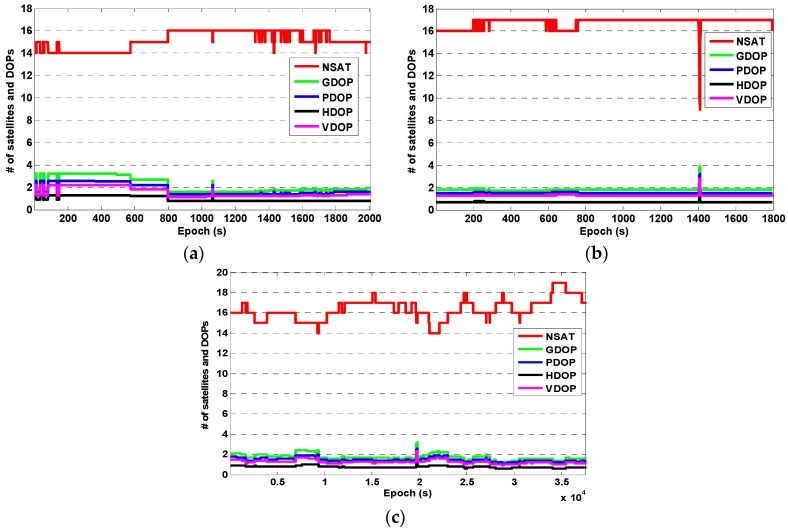
The number of visible satellites and the corresponding DOPs in the static test: (**a**) experiment S1; (**b**) experiment S2; and (**c**) experiment S3.

In the static test, experiments S1 and S2 were carried out on an urban road on 31 January 2016 and experiment S3 was carried out at the Academy of Opto-Electronics on 2 November 2016. The testing periods were about 35 min for S1, 30 min for S2, and 10.5 h for S3, respectively. The approximate locations of the user in each experiment are marked on the map shown in Figure 5. The number of visible BDS + GPS satellites (referred to as NSAT) and the corresponding dilution of precision (DOP), i.e., geometric DOP (GDOP), position DOP (PDOP), horizontal DOP (HDOP), and vertical DOP (VDOP), for experiments S1, S2, and S3 are shown in Figure 6, respectively. It can be seen that the number of visible BDS + GPS satellites in each experiment was about 16, and for most of the epochs the PDOP was smaller than 2.0 and the HDOP was about 1.0. It is worth noting that the dramatic drop of about seven satellites for three seconds in experiment S2 (Figure 6b) was due to the blockage of GNSS signals at one side which was caused by a very large vehicle passing by our experimental car.

**Figure 7:**
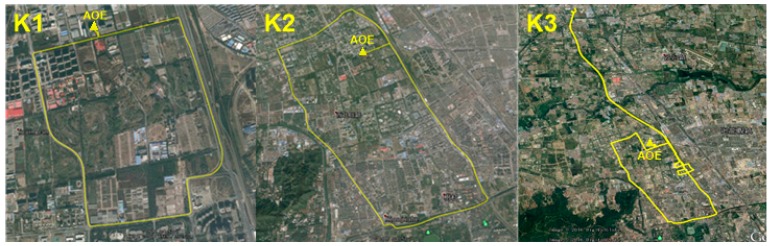
The vehicle trajectories of kinematic experiments K1, K2, and K3.

**Figure 8:**
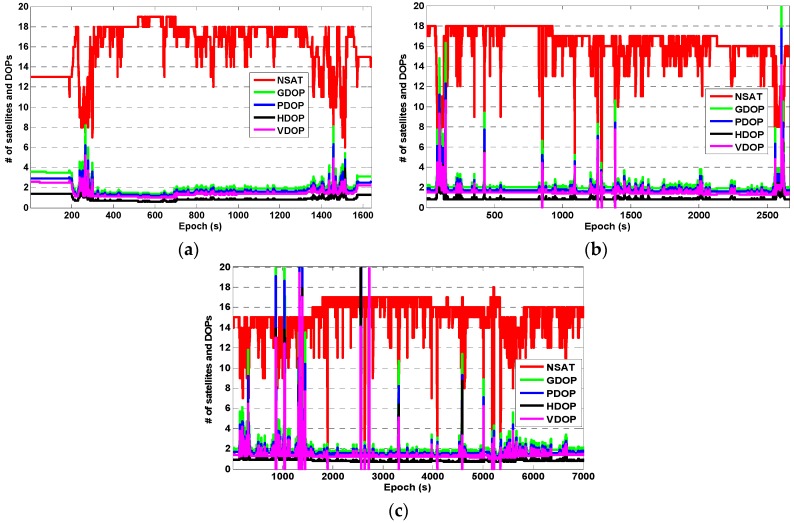
The number of visible satellites and the corresponding DOPs in the kinematic test: (**a**) experiment K1; (**b**) experiment K2; and (**c**) experiment K3.

In the kinematic test, experiments K1 and K2 were carried out on 1 February 2016, and experiment K3 was carried out on 3 November 2016. The testing periods were about 25 min for K1, 45 min for K2, and 2 h for K3, respectively. The vehicle speed was about 50–80 km/h and the trajectories of each experiment are shown in Figure 7. The experimental routes covered the urban road and the highway. The longest distance (baseline) between the rover and the reference station was about 16 km (in K3). The number of visible BDS + GPS satellites and the corresponding DOPs for each experiment are shown in Figure 8. It can be seen that when the experimental vehicle drove on the road, the number of visible satellites and the corresponding DOPs varied significantly due to the obstructions caused by high-rise buildings, street canyons, and overhead viaducts, etc. When under good observation conditions, the number of visible satellites can reach 14–18. However, it declined to less than 10 (even less than four for a few epochs) visible satellites when the occlusions occurred and the DOPs of many epochs became dramatically larger due to the poor geometric distribution of the visible satellites.

For all of the experiments, (1) the ambiguity fixed-rate was defined as the percentage of the ambiguity-fixed epochs to the whole testing period; (2) the fixed solutions mean that those positioning results were obtained with ambiguity parameters being fixed into integers; and (3) when the communication to the DPC failed for receiving the differential corrections, the solutions were derived from single-point positioning.

### 3.2. Results

#### 3.2.1. Static Test

**Figure 9:**
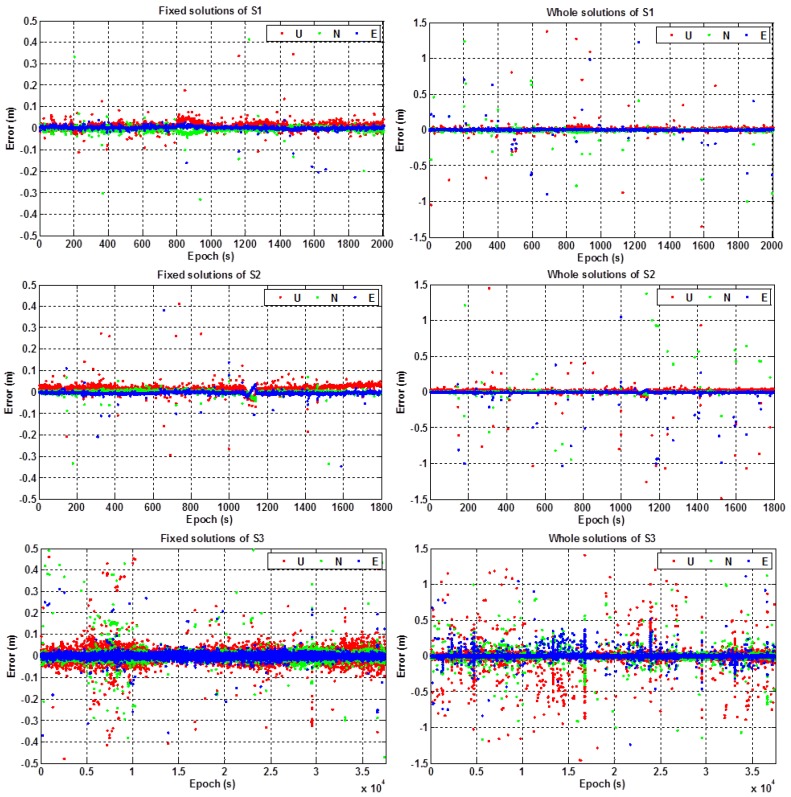
Positioning errors of experiment S1 (**top**), S2 (**middle**), and S3 (**bottom**) in the static test.

Figure 9 shows the positioning errors of the fixed solutions and the whole solutions in the east (E), north (N), and up (U) components for the three static experiments S1, S2, and S3, respectively. Table 2 illustrates the performances of ambiguity resolution and also the results of RMS value of the positioning errors in the horizontal (H) and vertical (V) components. In addition, Table 3 gives the percentages of the positioning errors at different levels, i.e., within 0.1 m, 0.2 m, 0.5 m, 1.0 m and larger than 1.0 m, for the static experiments S1, S2, and S3, respectively.

From Figure 9 and Table 2 and Table 3, we can see that: (1) the ambiguity fixed-rate of RTK positioning was about 97% on average for the static test; (2) the positioning errors of the fixed solutions were within 10 cm for most of the experimental periods, and the positioning errors of the unfixed solutions were within 1.5 m for most of the testing periods; (3) the positioning accuracy (RMS) of the RTK fixed solutions can achieve better than 3 cm in the horizontal component and 4 cm in the vertical component; (4) the percentages of the positioning errors that were within 0.5 m can reach up to more than 98% (both for the horizontal and vertical components) for the three static experiments; and (5) the positioning accuracy (RMS) of the whole solutions were better than 0.15 m and 0.25 m in the horizontal and the vertical components, respectively.

#### 3.2.2. Kinematic Test

**Figure 10:**
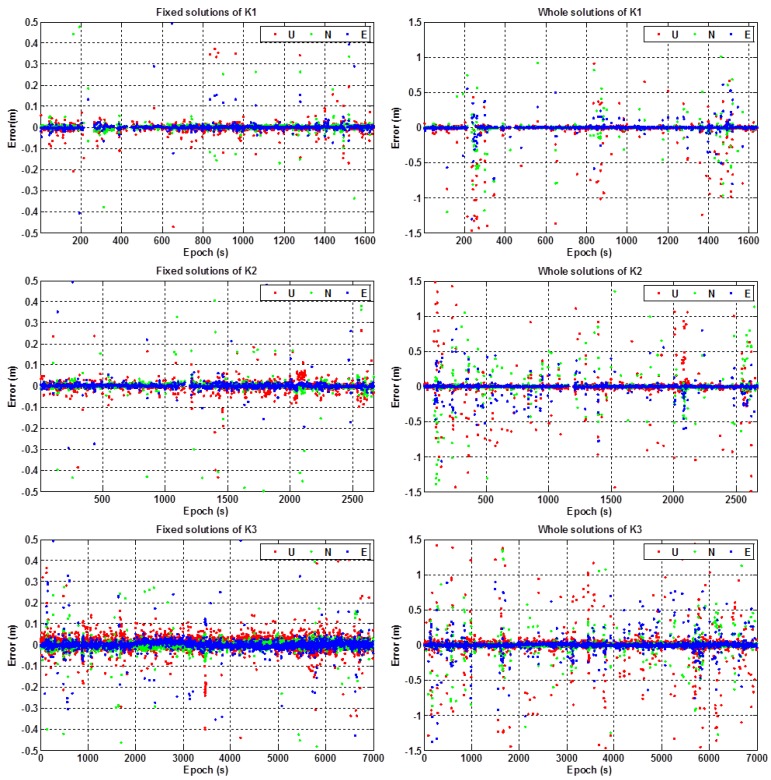
Positioning errors of experiment K1 (**top**), K2 (**middle**), and K3 (**bottom**) in the kinematic test.

Figure 10 shows the positioning errors of the fixed solutions and the whole solutions in the east, north, and up components for the three kinematic experiments of K1, K2, and K3, respectively. Table 4 shows the performances of ambiguity resolution and the results of the RMS values of positioning errors in the horizontal and vertical components. In addition, Table 5 gives the percentages of the positioning errors at different levels, i.e., within 0.1 m, 0.2 m, 0.5 m, 1.0 m and larger than 1.0 m, for the kinematic experiments K1, K2, and K3, respectively.

It can be seen from Figure 10 and Table 4 and Table 5 that: (1) the ambiguity fixed-rate of RTK positioning was better than 90% for the three kinematic experiments, which was a little lower than that of the static test; (2) as with the static test, the positioning errors of the fixed solutions were within 10 cm for most of the testing periods, and the positioning errors of the unfixed solutions were within 1.5 m for most of the testing periods; (3) the positioning accuracy (RMS) of the RTK fixed solutions on average can achieve about 10 cm in the horizontal component and 13 cm in the vertical component; (4) the percentages of the positioning errors that were within 0.5 m can reach up to more than 90% (both for the horizontal and vertical components) for the three kinematic experiments; and (5) the positioning accuracy (RMS) of the whole solutions during the testing periods was better than 0.30 m in the horizontal component and 0.45 m in the vertical component on average, respectively.

## 4. Conclusions

In this work, a feasible solution for achieving RTK positioning with sub-meter-level accuracy with a smart device and a low-cost GNSS terminal was proposed and a set of user-side application software for BDS/GNSS RTK positioning based on the Android platform was developed from scratch. The performance of the proposed solution was validated by static and kinematic BDS/GPS combined single-epoch RTK positioning in a real urban environment.

The experimental results show that: (1) for the static test, the ambiguity fixed-rate of RTK positioning was about 97%, and the positioning RMSs were better than 0.15 m in the horizontal component and 0.25 m in the vertical component, respectively; (2) for the kinematic test, whose performance was slightly worse than that of the static test, the ambiguity fixed-rate was better than 90%, and the positioning RMSs were better than 0.30 m in the horizontal component and 0.45 m in the vertical component, respectively; (3) the percentages of the positioning errors that were within 0.5 m (for the horizontal and vertical components) can reach up to more than 98% for the static test and 90% for the kinematic test. Therefore, the performance of the smart device–supported BDS/GNSS RTK positioning proposed and developed in this work has the potential to meet the demands of urban LBS with sub-meter precision.

In addition, future works will be mainly focused on: (1) the integration of the external low-cost GNSS module and the smart device for a complete positioning and navigation terminal and its real application in urban high-precision LBS; (2) the realization of network RTK (NRTK) positioning using a network of reference stations for larger coverage and higher accuracy; and (3) the possibility and potential for supporting real-time PPP and PPP-RTK positioning.

## Figures and Tables

**Table 1:** The performances of RTK positioning with different latency times of the differential corrections.

Validation Approaches	Latency Time of Corrections (s)	Ambiguity Fixed-Rate (%)	RMS Error (cm)		
			Fixed Solutions	Unfixed Solutions	Whole Solutions
Traditional RT with fixed threshold (=2.0)	0	96.9	1.5	40.7	9.0
	5	95.5	1.7	46.7	10.2
	10	91.0	2.0	50.8	12.4
FF-RT with tolerable failure-rate (=0.01)	0	100	1.7	—	1.7
	5	99.8	4.3	36.8	6.1
	10	99.7	5.3	38.4	8.2

**Table 2:** The performances of RTK positioning in the static test.

Experiments	Ambiguity Fixed-Rate (%)	RMS (m)					
		Fixed Solutions		Unfixed Solutions		Whole Solutions	
		H	V	H	V	H	V
S1	97.8	0.03	0.04	0.45	1.24	0.14	0.25
S2	98.1	0.02	0.04	0.67	0.84	0.15	0.21
S3	95.7	0.02	0.03	0.25	0.43	0.07	0.09

**Table 3:** Percentages of positioning errors at different levels for the static test.

Experiments	Error ≤ 0.1 m (%)		Error ≤ 0.2 m (%)		Error ≤ 0.5 m (%)		Error ≤ 1.0 m (%)		Error > 1.0 m (%)	
	H	V	H	V	H	V	H	V	H	V
S1	98.26	98.10	98.57	98.46	99.13	98.72	99.54	99.03	0.46	0.97
S2	97.59	97.30	98.05	97.70	98.39	98.45	99.14	99.02	0.86	0.98
S3	97.69	97.61	98.56	98.11	99.22	98.97	99.51	99.40	0.49	0.61

**Table 4:** The performances of RTK positioning in the kinematic test.

Experiments	Ambiguity Fixed-Rate (%)	RMS (m)					
		Fixed Solutions		Unfixed Solutions		Whole Solutions	
		H	V	H	V	H	V
K1	92.4	0.08	0.12	1.07	1.25	0.24	0.40
K2	90.6	0.09	0.13	1.15	1.78	0.28	0.47
K3	93.5	0.11	0.14	1.23	1.42	0.32	0.45

**Table 5:** Percentages of positioning errors at different levels for the kinematic test.

Experiments	Error ≤ 0.1 m (%)		Error ≤ 0.2 m (%)		Error ≤ 0.5 m (%)		Error ≤ 1.0 m (%)		Error > 1.0 m (%)	
	H	V	H	V	H	V	H	V	H	V
K1	80.30	80.75	85.51	84.61	93.50	90.41	97.30	94.53	2.70	5.47
K2	85.42	85.66	86.98	87.46	92.53	90.80	96.67	94.09	3.33	5.91
K3	93.35	92.54	94.51	94.01	96.92	95.84	98.19	97.47	1.81	2.53
